# Pathology

**Published:** 1890-11-29

**Authors:** 


### PATHOLOGY.

Dr. Savill in commencing his lecture said a few words upon what is known concerning the motor centres and motor tracts of the brain. The motor area is the region corresponding to that part of the brain lying behind and in front of the fissure of Rolando. It has been mapped out experimentally by the electrical stimulation of these regions. Tracing the various motor centres from below upwards, and before backwards, they occur in the following order: face, arm, shoulder, leg, the trunk centre being situated at the top and rather on the internal surface, that is to say at the marginal convolution. The fibres from all of these centres join in one large band, the fibres from the trunk being situate behind, and thus their order from before backwards is face, arm, leg, trunk. They then pass through the posterior part of the internal capsule. This may be divided into four regions corresponding to the bundles above mentioned, the order from before backwards being face, arm, leg, trunk. From the internal capsule the motor bundles pass through the pyramid, and here about three-quarters pass to the opposite side, continue down as the indirect pyramidal tract; the remaining fibres pass down the same side in the direct pyramidal tract. The case of A. H., a woman aged 63, was then described. From her family history it was gathered that her father and mother both lived to old age. Two of her children and an older sister were insane. She married young, and enjoyed good health, but had been a heavy drinker, more or less, all her life, and was stated to have had delirium tremens. On November 10 of the present year, while sitting in a chair, it was noticed that her mouth was drawn to the right, and that the food which was present in her mouth could not be swallowed. There was no loss of consciousness, no convulsions, but the urine was passed involuntarily. She was placed in bed, and the next day revealed some slight paralysis of the left side of the face. On the 12th weakness of the left side and arm was noticed, and her mental condition which had been somewhat emotional became more so. On the 16th she could only read a few letters when directed to, and if asked, revealed symptoms of pain in the left parietal eminence. On the 17th it was noticed she had a tendency to turn her head towards the right. Finally, the left leg became also paralysed. The pulse was full, the arteries dilated, she became apathetic, and died comatosed on the tenth day after the seizure. Post-mortem it was found that the left ventricle of the heart was slightly hypertrophed, and there was slight atheroma at the commencement of the aorta. Microscopically a granular degeneration of the muscle fibres of the heart was found. The lungs were fibrotic, with evidences of old bronchial mischief. The liver weighed thirty-five ounces, and there was some mottling of its substance. Microscopically, there was slight cirrhosis, together with marked fatty changes in the periphery of the lobules. The spleen was normal, the kidneys weighed 4 and 4½ oz. respectively, their substance was firm, and the capsules did not readily strip off. The vessels of the brain were markedly atheromatous, and the substance of the brain presented several lesions of various dates. The right centrum ovale was the seat of softening, which occupied the anterior two-thirds of the white substance, and extended through the internal capsule. As to the nature of this lesion it was probably a softening due to blocking of the vessels supplying it. The points in the differential diagnosis between hæmorrhage and softening, are as follow:—One, as to the mode of advent, softening is gradual, hæmorrhage is sudden; two, in softening the onset occurs usually without convulsions and without loss of consciousness; three, in softening there is, as a rule, no rigidity as occurs in hæmorrhage.

